# Phosphoinositide 3-kinase: friend and foe in cardiovascular disease

**DOI:** 10.3389/fphar.2015.00169

**Published:** 2015-08-13

**Authors:** Alessandra Ghigo, Mingchuan Li

**Affiliations:** Molecular Biotechnology Center, Department of Molecular Biotechnology and Health Sciences, University of Torino, Torino, Italy

**Keywords:** phosphoinositide 3-kinase, heart failure, atherosclerosis, inflammation, scaffold proteins, cross-talk

## Abstract

Class I phosphoinositide 3-kinases (PI3Ks) are a family of lipid kinases activated by cell membrane receptors, either receptor tyrosine kinases (RTKs) or G protein–coupled receptors (GPCRs), to catalyze the production of the lipid second messenger phosphatidylinositol (3,4,5)-trisphosphate (PIP3). These enzymes engage multiple downstream intracellular signaling pathways controlling cell proliferation, survival and migration. In the cardiovascular system, the four class I PI3K isoforms, PI3Kα, PI3Kβ, PI3Kδ, and PI3Kγ are differentially expressed in distinct cell subsets which include cardiomyocytes, fibroblasts, endothelial, and vascular smooth muscle cells as well as leukocytes, suggesting specific functions for distinct PI3K isoenzymes. During the last decades, genetic disruption studies targeting different PI3K genes have elucidated the contribution of specific isoenzymes to cardiac and vascular function regulation, highlighting both beneficial and maladaptive roles. New layers of complexity in the function of PI3Ks have recently emerged, indicating that distinct PI3K isoforms are interconnected by various crosstalk events and can function not only as kinases, but also as scaffold proteins coordinating key signalosomes in cardiovascular health and disease. In this review, we will summarize major breakthroughs in the comprehension of detrimental and beneficial actions of PI3K signaling in cardiovascular homeostasis, and we will discuss recently unraveled cross-talk and scaffold mechanisms as well as the role of the less characterized class II and III PI3K isoforms.

## Introduction

Phosphoinositide 3-kinases (PI3Ks) are a family of lipid and protein kinases that primarily function by catalyzing the phosphorylation of D3 position on the inositol ring of phosphatidylinositols (PtdIns). Depending on the nature of phosphorylated products, PI3Ks are currently classified into three classes.

Class I PI3Ks catalyze the production of phosphatidylinositol (3,4,5)-trisphosphate [PtdIns(3,4,5)P_3_ or PIP_3_] from phosphatidylinositol (4,5)-bisphosphate [PtdIns(4,5)P_2_]. These enzymes are obligate heterodimers composed by a p110 catalytic subunit among the four identified p110α, β, γ, and δ isoforms, and an adaptor subunit of either p85 or p101/p84 family. Class I PI3Ks can be further subgrouped into class IA and IB, according to the identity of the regulatory subunit as well as the type of membrane activating receptor. Class IA PI3Ks, PI3Kα, β, and δ, associate with p85 adaptors and are primarily triggered by tyrosine kinase receptors (RTKs), while class IB PI3Kγ binds to either p101 or p84 subunit and has long been considered to respond only to G protein–coupled receptor (GPCR) agonists (Wymann et al., 2003). However, the classical view has been recently modified by the finding that PI3Kβ can also signal downstream of GPCRs (Ciraolo et al., 2008; Guillermet-Guibert et al., 2008) and p110γ can be engaged by RTKs in a Ras/p84-dependent way (Schmid et al., 2011).

Following class I PI3K activation, PIP_3_ accumulates at the plasma membrane and recruits a group of pleckstrin homology (PH) domain-containing proteins, including phosphoinositide-dependent kinase-1 (PDK1) and protein kinase B (Akt/PKB). PIP_3_-activated PDK1 in turn phosphorylates Akt at Thr308 site that, together with Ser473 phosphorylation by mammalian target of rapamycin complex 2 (mTORC2), ensures full Akt activation. This eventually triggers multiple downstream signaling pathways involved in protein synthesis, cell proliferation, metabolism and survival (Franke, 2008).

PI3K activity is counteracted by PTEN (phosphatase and tensin homolog deleted from chromosome 10), the major endogenous PI3K inhibitor, which dephosphorylates PIP_3_ on the D3 position. PI3Ks and PTEN thus keep the balance of cellular PIP_3_ levels that, when deregulated by either amplification of PI3Ks or loss of PTEN, may lead to dramatic consequences, such as cell transformation (Cully et al., 2006).

Different from class I, class III includes only one member, Vps34, which is critically involved in the regulation of vesicular trafficking and autophagy via PtdIns(3)P generation (Funderburk et al., 2010). Conversely, little information is available about the function of class II PI3Ks, including PI3K-C2α, PI3K-C2β and PI3K-C2γ. PI3K-C2α and PI3K-C2γ are the best characterized isoforms and the emerging view is that they also critically contribute to vesicular trafficking by regulating the production of PtdIns(3)P (Franco et al., 2014) and PI(3,4)P2 (Braccini et al., 2015) pools.

**FIGURE 1:**
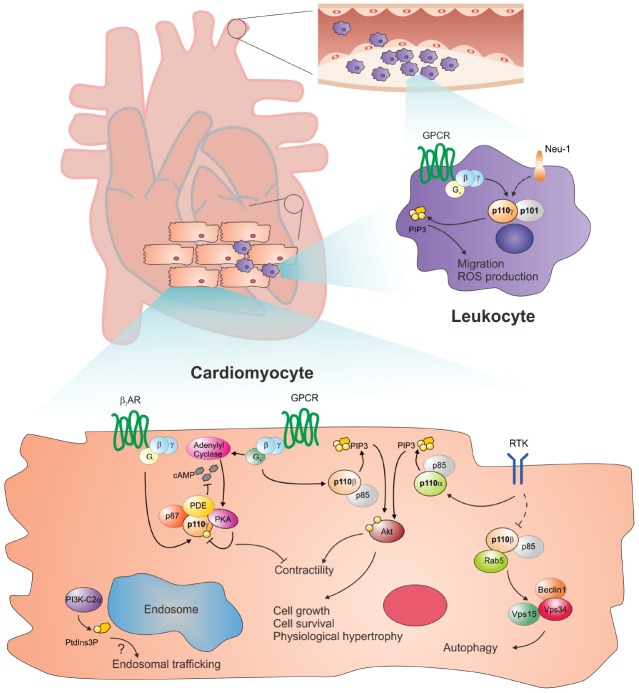
**Distinct PI3K isoenzymes control specific cardiovascular processes.** Class I PI3Kγ is enriched in immune cells and primarily controls leukocyte infiltration in the myocardium and arteries, a key pathogenic event in both heart failure and atherosclerosis. Following receptor activation, PI3Kγ catalyzes the production of the lipid second messenger PIP3 which in turn promotes leukocyte migration and ROS production. On the other hand, cardiomyocyte PI3Kγ is a dual face enzyme which negatively regulates β-AR/cAMP signaling during the natural history of heart failure, via kinase-dependent and independent events. By serving as an A-kinase anchoring protein (AKAP), PI3Kγ tethers major PDE isoforms near their activator PKA. PI3Kγ-bound PKA phosphorylates and activates PDEs, which in turn shape discrete microdomains of cAMP/PKA action, ultimately depressing cardiac contractility. In physiological conditions, PI3Kγ lipid kinase activity is inhibited by PKA, but escapes PKA-mediated blockade in failing hearts where it critically contributes to PIP3-dependent β-AR internalization. Conversely, the other major cardiac PI3K isoform, PI3Kα, is engaged by RTKs and provides positive control of myocardial contractility and hypertrophy via the PIP3/Akt classical pathway in response to diverse stimuli, such as pressure overload and myocardial infarction. Class I PI3Kβ instead appears to function not only as a kinase downstream GPCRs and RTKs, but also as a scaffold protein, which associates with Rab5 within the Rab5-Vps34-Beclin complexes to activate cardiac autophagy. Finally, the recently characterized class II PI3K-C2α governs local PtdIns(3)P production and likely participates to endosomal trafficking within the cardiovascular system, through yet unexplored mechanisms.

Different PI3K isoforms show peculiar expression patterns, with PI3Kα being ubiquitously expressed and enriched in cardiomyocytes, and PI3Kγ functioning in both leukocytes and cardiac cells. This implies that distinct PI3K isoenzymes participate to specific key processes in the maintenance of cardiovascular homeostasis, including cardiomyocyte hypertrophy and contractility as well as myocardial and vascular inflammation (Figure 1). In this review, we will summarize major breakthroughs in the comprehension of detrimental and beneficial actions of PI3K isoenzymes in cardiovascular health and disease, and we will discuss recently unraveled cross-talk and scaffold mechanisms as well as the role of the less characterized class II and III PI3K isoforms.

## PI3Kα: Essential Role in Cardiac Growth and Contractility

In the cardiovascular system, PI3Kα is activated by multiple RTKs. In cardiomyocytes, PI3Kα is engaged by insulin or insulin-like growth factor-1 (IGF-1) to regulate cardiac growth and development. Furthermore, fibroblast growth factor (FGF), platelet-derived growth factor (PDGF) and vascular endothelial growth factor (VEGF) can activate PI3Kα in endothelial cells, fibroblasts and vascular smooth muscle cells.

In the last decade, studies based on genetic modified animals have revealed a critical role for PI3Kα-Akt cascade in promoting cardiomyocyte postnatal growth and survival. Cardiac-restricted over-expression of either PI3Kα (Shioi et al., 2000) or its upstream signaling IGF-1 receptor (IGF1R; McMullen et al., 2004) results in increased cardiomyocyte size and larger hearts. In contrast, expression of dominant-negative p110α or deletion of PI3Kα regulatory subunit p85 leads to reduced heart size (Shioi et al., 2000; Crackower et al., 2002; Luo et al., 2005) and abolishes IGF1R overexpression-induced physiological hypertrophy (McMullen et al., 2004). Consistent with its key role as endogenous PI3K inhibitor, PTEN inactivation in the heart recapitulates the phenotype observed in PI3Kα overexpressing animals (Crackower et al., 2002), suggesting a central role of PI3Kα-dependent PIP3 in regulating heart growth. Similarly, Akt genetic manipulations phenocopy PI3Kα alterations. Constitutive activation of Akt increases cardiomyocyte size and leads to concentric left ventricle hypertrophy, while Akt knockout mice display smaller hearts (Condorelli et al., 2002; Matsui et al., 2002; DeBosch et al., 2006).

In keeping with a crucial role in cardiac postnatal growth, PI3Kα promotes physiological hypertrophy and sustains systolic function in adults. Notably, PI3Kα is protective also in contexts of heart disease. In a murine model of dilated cardiomyopathy (DCM), PI3Kα activation mimics the beneficial effects of exercise training by limiting fetal gene reprogramming and ultimately prolonging survival, while dominant-negative PI3Kα mice show reduced lifespan (McMullen et al., 2007). Moreover, ablation of PI3Kα exacerbates pathological hypertrophy triggered by either pressure overload or myocardial infarction, whereas PI3Kα hyperactivation results beneficial in both contexts (McMullen et al., 2007; Lin et al., 2010). Thus, PI3Kα is essential for physiological myocardial growth and protects the heart against pathological remodeling and failure.

Besides the control of cardiac hypertrophy, PI3Kα has been linked to the regulation of coronary angiogenesis, which is essential to provide adequate oxygen and nutrient supply to the myocardium. Studies demonstrate that PI3Kα and PI3Kδ, instead of other isoforms, are central to cardiovasculogenesis of embryonic stem (ES) cells, in response to VEGF stimulation (Bekhite et al., 2011). In embryos, PI3Kα deficiency results in vascular defects and diminished expression of tunica intima endothelial kinase 2 (Tie2), a receptor tyrosine kinase regulating vascular development (Lelievre et al., 2005). Accordingly, cardiac-specific activation of Akt promotes VEGF and angiopoietin-2 expression within cardiomyocytes via a mTOR-dependent mechanism, and ultimately increases myocardial capillary density, which is accompanied by physiological hypertrophic remodeling (Shiojima et al., 2005). Similarly, PTEN inactivation and Akt overexpression prevent the reduction of myocardial capillaries induced by pressure overload, thus indicating that preserved capillary density contributes to the protection against heart failure observed in these animals (Ceci et al., 2007; Oudit et al., 2008).

In addition to its involvement in myocardial viability and anabolism regulation, PI3Kα critically affects cardiac contractility. Cardiac-specific PI3Kα overexpression results in enhanced contractility in *ex vivo* Langendorff-perfused hearts (Yano et al., 2008), while overexpression of dominant-negative PI3Kα reduces basal contractility (Liang et al., 2010). Moreover, both PTEN-null and IGF-1-treated hearts show enhanced L-type Ca^2+^ currents (I_*Ca*,*L*_), while pharmacological inhibition of either PI3Kα or Akt reduces Ca^2+^ currents and contractility (Sun et al., 2006; Lu et al., 2009), pointing to a crucial role for PI3Kα in controlling cardiac contractile function. Mechanistically, PI3Kα modulates the expression of key components of the calcium-handling machinery, such as the L-type voltage-gated calcium channel (Cav1.2), the type-2 ryanodine receptor (RyR) and sarco(endo)plasmic reticulum calcium ATPase 2a (SERCA2a) (Yano et al., 2008; Lu et al., 2009). Intriguingly, a recent study reveals that PI3Kβ cooperates with PI3Kα to regulate cardiomyocyte structure and contractility (Wu et al., 2011). Of note, PI3Kα has also been shown to regulate atrial myocyte function. PI3Kα activity is significantly lower in patients with atrial fibrillation, the most common form of cardiac arrhythmia, than in controls with normal sinus rhythm. Furthermore, PI3Kα over-expression reduces atrial fibrosis and improves cardiac conduction in a murine model of dilated cardiomyopathy (Pretorius et al., 2009).

Overall, these works thus identify beneficial roles for PI3Kα in the maintenance of cardiovascular homeostasis, via regulation of cardiomyocyte growth, contractility and coronary angiogenesis.

## PI3Kγ: Integrating Leukocyte and Cardiomyocyte Signals During the Natural History of Heart Disease

Distinct from PI3Kα, PI3Kγ is not ubiquitously expressed but enriched in specific cell subsets, including leukocytes and cardiac cells. This implies a major role for this isoform in controlling not only cardiomyocyte pathobiology but also in orchestrating the inflammatory response associated with cardiovascular injury (Ghigo et al., 2014).

PI3Kγ expression is upregulated both in patients and in mouse models of atherosclerosis (Fougerat et al., 2008), a chronic disease where the artery wall thickens as a result of massive leukocyte infiltration, leading to fat plaque formation and ultimately to thrombosis and cardiac infarction. Genetic and pharmacological inhibition of PI3Kγ significantly dampens leukocyte recruitment, primarily macrophages and T cells, to the atherosclerotic lesion, and improves plaque stabilization in both apolipoprotein E-null (ApoE^–/–^) (Chang et al., 2007) and low-density lipoprotein receptor–deficient (LDLR^–/–^) mice (Fougerat et al., 2008). Notably, PI3Kγ-null (PI3Kγ^–/–^) bone marrow transplantation in LDLR^–/–^ mice fully recapitulates the protection of whole body PI3Kγ inactivation, thus demonstrating a crucial role for leukocyte PI3Kγ in the atherosclerotic process (Fougerat et al., 2008). Mechanistically, PI3Kγ participates to neuraminidase-1 (Neu-1) signaling downstream leukocyte elastin receptor complex (ERC) and crucially governs monocyte migration, ROS production and the ensuing atherosclerosis development in response to elastokines (Gayral et al., 2014). Finally, recent evidence suggests that the atheroprotective effects of PI3Kγ inhibition could be also explained by a major role of the enzyme in the control of macrophage proliferation, but not M1/M2 polarization, within atherosclerotic plaques (Zotes et al., 2013).

Besides playing a crucial role in atherogenesis, PI3Kγ-directed leukocyte infiltration is a major determinant of pressure overload-induced cardiac maladaptive remodeling. Mice expressing a knock-in catalytically inactive PI3Kγ (PI3Kγ-KD) or receiving a PI3Kγ-selective inhibitor display reduced fibrosis and preserved cardiac function up to 16 weeks after transverse aortic constriction (TAC). Intriguingly, wild-type animals carrying PI3Kγ-KD bone marrow are fully protected against TAC-induced dysfunction while, in the presence of a wild-type immune system, PI3Kγ-KD hearts display prominent leukocyte infiltration and fibrosis. Nevertheless, these latter chimeric mice display reduced left ventricular dilation and preserved contractile function compared to wild-type at later stages after TAC (Damilano et al., 2011), thus demonstrating that not only leukocyte, but also cardiomyocyte PI3Kγ mediates detrimental effects in hypertensive heart disease.

In cardiomyocytes, PI3Kγ counteracts the positive inotropic action of PI3Kα by affecting β-adrenergic receptor (β-AR) signaling, a key regulator of myocardial contractility, through both protein and lipid kinase functions (Perino et al., 2011; Vasudevan et al., 2011). Notably, PI3Kγ lipid kinase activity is negligible in physiological conditions, owing to both low expression levels and protein kinase A (PKA)-dependent phosphorylation, but results dramatically upregulated under adrenergic stress, such as that occurring in congestive heart failure (Perino et al., 2011). In this setting, PI3Kγ escapes PKA-mediated inhibition and critically contributes to the pathological decrease in myocardial β-AR density. PI3Kγ indeed cooperates with GPCR kinase-2 [GRK-2, also known as β-adrenergic receptor kinase-1 (β-ARK-1)], a key biomarker molecule upregulated in human heart failure, that by mediating receptor tail phosphorylation and the following binding by β-arrestin, interrupts G-protein coupling and initiates the process of internalization (Woodall et al., 2014). In keeping with these findings, genetic ablation of PI3Kγ and administration of a selective PI3Kγ inhibitor both significantly normalize β-AR density and improve compromised cardiac contractility of failing hearts (Perino et al., 2011).

Overall, these works unveil a key role for both leukocyte and cardiomyocyte PI3Kγ in the development of heart failure and provide proof-of-concept that pharmacological targeting of PI3Kγ activity represents a unique opportunity to treat cardiovascular diseases through combined actions on both inflammatory and heart cells.

## Class I PI3Ks: Scaffold Roles and Cross-talk Networks

Although compelling evidence points to key roles of PI3K kinase activity in cardiac pathophysiology, a new layer of complexity in the regulation of these enzymes has established in the last decade, indicating that these proteins function not only as kinases but also as scaffold proteins (Hirsch et al., 2009). PI3Kγ represents the prototypical PI3K isoenzyme with adaptor activity, exerting kinase-unrelated functions not only in the myocardium, but also in other districts including endothelial progenitors (Madeddu et al., 2008) and neurons (D’Andrea et al., 2015). In the healthy heart, PI3Kγ acts as an A-kinase anchoring protein (AKAP) rather than a kinase, tethering major PDE isoforms near their activator PKA (Perino et al., 2011; Ghigo et al., 2012). In this context, PI3Kγ-bound PKA phosphorylates and activates PDE3 and PDE4, eventually shaping discrete cAMP microdomains and initiating defined sets of PKA-mediated events downstream of β_2_-ARs. Accordingly, PKA-dependent phosphorylation of key Ca^2+^ handling proteins, a crucial event in the regulation of cardiac excitation-contraction coupling (Santulli and Marks, 2015), is dramatically altered in absence of PI3Kγ. L-type calcium channel and phospholamban are hyperphosphorylated in PI3Kγ^–/–^ cardiomyocytes and ultimately lead to spontaneous Ca^2+^ release events and arrhythmic Ca^2+^ transients. Intriguingly, PI3Kγ-directed protein complexes are functionally impaired in heart failure, a condition where ventricular arrhythmia is a major cause of death (Perino et al., 2011; Ghigo et al., 2012). Thus, deregulation of PI3Kγ scaffold function appears an important component of heart failure-related arrhythmias.

Besides PI3Kγ, also PI3Kβ, the other GPCR-activated class I PI3K isoform, has been reported to exert roles that are independent of its catalytic activity (Hirsch et al., 2009). In murine embryonic fibroblasts (MEFs), genetic elimination of either PI3Kβ expression or kinase activity results in defective internalization of the transferrin receptor (TfR) and the epidermal growth factor receptor (EGFR; Ciraolo et al., 2008; Jia et al., 2008). This involves a protein–protein interaction mechanism, likely relying on the ability of PI3Kβ to associate and activate the small GTPase Rab5, a key regulator of the endocytic machinery (Ciraolo et al., 2008). Whether the scaffold activity of PI3Kβ affects endocytic trafficking within cardiomyocytes is still unexplored. However, recent evidence unveils a crucial role for PI3Kβ-mediated activation of Rab5 in cardiac autophagy induction (Dou et al., 2010, 2013). This implies that PI3Kβ participates to the autophagic process as part of the Rab5-Vps34-Beclin complex, thereby favoring Vps34 kinase activity and PtdIns(3)P production, which in turn is essential for autophagosome formation. In keeping with this finding, starvation-induced autophagy is dramatically impaired in PI3Kβ-null (PI3Kβ^–/–^) hearts. Furthermore, the fact that loss of PI3Kβ fully prevents autophagy during pressure overload indicates that PI3Kβ-dependent control of autophagy may be also relevant to conditions of heart failure (Dou et al., 2010).

A further level of complexity in PI3K signaling regulation is provided by the intricate network of cross-talks connecting different PI3K isoenzymes, downstream of distinct cell surface receptors. Recent evidence highlights a strict cooperation between the two GPCR-activated class I PI3K isoforms, PI3Kβ and PI3Kγ, in the control of the sympathetic drive in the central nervous system downstream of the melanocortin 4 receptor (MC4R). Accordingly, both genetic and pharmacological simultaneous inhibition of these two isoforms result in reduced Akt-mediated activation of PDE3B and enhanced cAMP accumulation within the hypothalamus and the intermediolateral nucleus, ultimately impinging on white adipose tissue lipolysis (Perino et al., 2014). Whether PI3Kβ- and PI3Kγ-dependent adrenergic firing also affects cardiac function via neuron-cardiomyocyte crosstalk mechanisms is unclear and further studies are required to clarify this issue. On the other hand, compelling evidence establishes a tight connection between cardiomyocyte PI3Kγ and the other major isoform in the myocardium, PI3Kα. For instance, PI3Kγ has been reported to synergize with PI3Kα signaling in the control of cardiac hypertrophy. Mechanistically, PI3Kγ inhibits GSK-3 activation downstream of the insulin-PI3Kα-Akt pathway, through a kinase-independent mechanism that primarily prevents the interaction between the GSK-3 phosphatase, PP2A, and its activator, PP2A methyltransferase (PPMT-1). This eventually results in enhanced PI3Kα downstream signaling and cardiac hypertrophy induction (Mohan et al., 2013). In keeping with a cardioprotective role of this PI3Kα/PI3Kγ synergy, compound deletion of PI3Kα and PI3Kγ genes in the myocardium results in severe age-dependent cardiomyopathy, while single mutant mice have preserved systolic and diastolic function up to 1 year of age (Zhabyeyev et al., 2014).

These works thus emphasize the need of re-thinking the classical paradigm of PI3K-Akt signaling by considering the growing multifaceted roles of class I PI3K isoforms in cardiovascular health and disease.

## The Emerging Role of Class II and III PI3K Isoforms

A further complication in the scenario of PI3K signaling comes from the recent characterization of less studied isoforms, including class II and III PI3Ks. The finding that mice lacking PI3K-C2α display defective cardiac looping and die at early embryonic stages highlights crucial developmental functions for this class II isoform (Yoshioka et al., 2012; Franco et al., 2014). New hints about its role in adult physiology are also emerging. Endothelial cell-specific PI3K-C2α deletion diminishes the number of PI(3)P-enriched endosomes, impairs vesicular trafficking and ultimately leads to defective delivery of VE-cadherin to endothelial cell junctions and defective junction assembly. Accordingly, PI3K-C2α disruption correlates with impaired vascular barrier integrity and higher incidence of dissecting aortic aneurism formation in response to angiotensin II infusion. These findings thus unveil PI3K-C2α as a novel intriguing therapeutic target for vascular diseases (Yoshioka et al., 2012). Recent evidence highlights a major involvement for this enzyme also in thrombotic disorders. *In vivo* PI3K-C2α deficiency results in unstable thrombi formation, leading to a significant increase in spontaneous thromboembolism and intermittent vascular reperfusion in a model of electrolytic injury of the carotid artery. This phenotype stems from the ability of PI3K-C2α to modulate internal membrane structure and, eventually, shear-dependent adhesion of platelets (Mountford et al., 2015). On the other hand, the cardiovascular impact of other class II isoforms, PI3K-C2β and PI3K-C2γ, is still mysterious and awaits further investigation. The role of the unique member of class III PI3Ks in cardiac pathobiology is instead emerging. In cardiomyocytes, Vps34 critically contributes to both endocytic and autophagic degradation. Cardiomyocyte-specific genetic deletion of Vps34 impairs starvation-induced autophagosome formation and leads to cardiomegaly and contractile dysfunction, unveiling an indispensable role for class III PI3K in normal cardiac function (Jaber et al., 2012). Nonetheless, our understanding of Vps34 kinase activity is still incomplete and further studies are required to clarify this issue.

## Conclusion

Altogether, these studies underscore both adaptive and maladaptive actions of PI3K signaling in cardiovascular homeostasis, with class I PI3Kα having prominent beneficial effects, including enhanced physiological hypertrophy and contractility, and PI3Kγ mediating detrimental signals leading to β-AR cascade inhibition. The reasons of this dichotomy are still not fully understood, but the growing hypothesis is that PI3K-associated beneficial and deleterious effects depend on specific upstream/downstream effectors and on the subcellular compartmentalization of PI3Ks. This highlights the critical need of elucidating the complexity of these central signaling pathways to rationally exploit PI3K hubs for therapeutic intervention design. The recent discovery of endomembrane-associated isoenzymes and of new isoform-specific interactors/effectors indicates that efforts are accumulating in this direction, but further investigation is required to validate the clinical use of PI3K modulators. This is urgent given that broad spectrum PI3K inhibitors are starting to emerge as potential new chemotherapeutic agents, although their cardiovascular use, efficacy and safety remain unclear.

### Conflict of Interest Statement

The authors declare that the research was conducted in the absence of any commercial or financial relationships that could be construed as a potential conflict of interest.
